# Non-photochemical Quenching Plays a Key Role in Light Acclimation of Rice Plants Differing in Leaf Color

**DOI:** 10.3389/fpls.2016.01968

**Published:** 2017-01-10

**Authors:** Xia Zhao, Tingting Chen, Baohua Feng, Caixia Zhang, Shaobing Peng, Xiufu Zhang, Guanfu Fu, Longxing Tao

**Affiliations:** ^1^State Key Laboratory of Rice Biology, China National Rice Research InstituteHangzhou, China; ^2^National Key Laboratory of Crop Genetic Improvement, MOA Key Laboratory of Crop Ecophysiology and Farming System in the Middle Reaches of The Yangtze River, College of Plant Science and Technology, Huazhong Agricultural UniversityWuhan, China; ^3^Zigong Institute of Agricultural SciencesZigong, China

**Keywords:** heat dissipation, light intensity, oxidative stress, photoinhibition, rice (*Oryza sativa* L.)

## Abstract

Non-photochemical quenching (NPQ) is an important photoprotective mechanism in rice; however, little is known regarding its role in the photosynthetic response of rice plants with differing in leaf color to different irradiances. In this study, two rice genotypes containing different chlorophyll contents, namely Zhefu802 (high chlorophyll) and Chl-8 (low chlorophyll), were subjected to moderate or high levels of light intensity at the 6-leaf stage. Chl-8 possessed a lower chlorophyll content and higher chlorophyll a:b ratio compared with Zhefu802, while Pn, F_v_/F_m_, and Φ_PSII_ contents were higher in Chl-8. Further results indicated that no significant differences were observed in the activities of Rubisco, Mg^2+^-ATPase, and Ca^2+^-ATPase between these genotypes. This suggested that no significant difference in the capacity for CO_2_ assimilation exists between Zhe802 and Chl-8. Additionally, no significant differences in stomatal limitation were observed between the genotypes. Interestingly, higher NPQ and energy quenching (qE), as well as lower photoinhibitory quenching (qI) and production of reactive oxygen species (ROS) was observed in Chl-8 compared with Zhefu802 under both moderate and high light treatments. This indicated that NPQ could improve photosynthesis in rice under both moderate and high light intensities, particularly the latter, whereby NPQ alleviates photodamage by reducing ROS production. Both zeaxanthin content and the expression of *PsbS1* were associated with the induction of NPQ under moderate light, while only zeaxanthin was associated with NPQ induction under high light. In summary, NPQ could improve photosynthesis in rice under moderate light and alleviate photodamage under high light via a decrease in ROS generation.

## Introduction

Rice (*Oryza sativa* L.) is one of the most important food crops and is consumed by more than 3 billion people worldwide (Fageria, 2007). Generally, rice is cultivated in regions with high light intensity where photosynthetic photon flux density is >2000 μmol m^−2^ s^−1^ at noon on sunny days. High light intensities saturate photosynthetic rates in the leaves of rice, and excess light can cause photoinhibition of photosystem II (PSII) resulting in a decrease in quantum yield and photosynthetic rate (Kramer et al., 2004; Kasajima et al., 2009). Photoinhibition has even been found to occur in rice growing under optimal conditions (Murchie et al., 1999). In order to mitigate photodamage, plants have developed several protective mechanisms, including non-photochemical quenching (NPQ), which harmlessly quenches the excitation of chlorophyll within the light-harvesting antennae of PSII by converting excitation energy into thermal energy, which can then be released (Kasajima et al., 2011).

The importance of NPQ for the protection of the photosynthetic apparatus is supported by its ubiquity in the plant kingdom (Niyogi and Truong, 2013). Mutants lacking the capacity to induce NPQ are more sensitive to photoinhibition (Dall'Osto et al., 2007; Allorent et al., 2013) and exhibit lower resistance to environment stressors, such as heat (Tang et al., 2007), drought (Cousins et al., 2002), low temperature (Xu et al., 1999), and salt (Neto et al., 2014). However, it has been reported that NPQ exerts an effect on the rate of PSII photochemistry by increasing the dissipation of excitation energy by non-radiative processes in the pigment matrices of PSII, which consequently results in a decrease in the efficiency of delivery of excitation energy for PSII photochemistry in low light conditions (Genty et al., 1990). In tropical environments, rice grows at light levels that may reach 2000 μmol m^−2^ s^−1^; an intensity level that can result in severe damage given that photosynthesis in rice saturates at intensities below 1000 μmol m^−2^ s^−1^ (Kasajima et al., 2011). According to one NPQ model (Harbinson, 2012; Zaks et al., 2012), rice leaves are often unable to use all the light absorbed by their photosynthetic pigments for CO_2_ fixation. A limited capacity for CO_2_ fixation limits photosynthetic electron transport, which then restricts the functioning of the reaction centers of photosystem I (PSI) and PSII. In the case of PSII, this results in side reactions that produce harmful singlet oxygens (Long et al., 2015) as well as damage to the reaction center (Evans and Caemmerer, 2011) and membranes (Davison et al., 2002).

Based on the kinetics of chlorophyll fluorescence relaxation in the dark, at least 3 components of NPQ have been distinguished: the energy dependent component, qE, which is triggered by the proton gradient across the thylakoid membrane and relaxes within seconds; a second component, qT, which depends on state transitions and relaxes within minutes; and a third component, qI, which is caused by photoinhibition and relaxes very slowly (Jahns and Holzwarth, 2012; Ruban and Murchie, 2012; Rochaix, 2014). The energy dependent qE is the major component of NPQ (Külheim et al., 2002). For qE, the formation of a ΔpH across the thylakoid membrane is the initial driving step. Acidification of the thylakoid lumen leads to the activation of the PsbS protein and the activation of violaxanthin de-epoxidase, which transforms violaxanthin through de-epoxidation first to antheraxanthin and then to zeaxanthin (Horton, 2014; Rochaix, 2014; Goss and Lepetit, 2015). The transformation of these xanthophylls, which are bound to the light harvesting complex (LHC) polypeptides, induces conformational changes in these proteins and triggers qE. It was proposed that zeaxanthin acts as an allosteric modulator in the thylakoid membrane, enhancing LHCII–proton-binding affinity (Demmig-Adams and Adams, 1996; Ware et al., 2014) whilst, PsbS increases the mobility of PSII membrane complexes, accelerating qE formation, and relaxation. Although the necessity of PsbS in the rapid induction of qE *in vivo* has been clearly demonstrated, it is dispensable under *in vitro* conditions as qE can be induced in the absence of PsbS when the pH gradient is sufficiently high (Johnson and Ruban, 2011). This indicates that direct protonation of thylakoid proteins can bypass the need for PsbS. Irrespective of the presence of the PsbS protein or zeaxanthin, the formation of NPQ requires a structural reorganization of the thylakoid membrane (Betterle et al., 2009). Recently, membrane reorganization was reported to occur in intact chloroplasts exposed to high light, resulting in clustered domains of LHCII and PSII reaction centers (Johnson and Ruban, 2011; Goral et al., 2012). Johnson et al. (2011) found that these structural changes occurred rapidly and reversibly within 5 min of illumination and dark relaxation, and were dependent on ΔpH; enhanced by the de-epoxidation of violaxanthin to zeaxanthin. In addition, numerous investigations have sought to identify further substances and mechanisms associated with non-photochemical quenching (Belgio et al., 2014; Wahadoszamen et al., 2014; Gupta et al., 2015; Leverenz et al., 2015).

The reaction center core complexes of PSI and PSII contain only chlorophyll a, while the LHC consists of chlorophyll a, chlorophyll b, and carotenoids. Chlorophyll b is present exclusively in the light-harvesting chlorophyll proteins LHCI and LHCII (Kura-Hotta et al., 1987). Therefore, the increase in the chlorophyll a:b ratio indicates the decrease in light-harvesting chlorophyll proteins relative to the reaction center complexes. In the shade, light is efficiently harvested in photosynthesis. However, in full sunlight there is much excessive light energy and it is vitally important to switch to specific antenna states and trigger NPQ (Pascal et al., 2005). This suggests that light acclimation contributes to the different photosynthetic properties of leaves. In contrast to sun-acclimated leaves, shade-acclimated leaves have a lower chlorophyll a:b ratio and relatively more chlorophyll in the LHCs (Evans, 1989; Lichtenthaler and Babani, 2004). The largest proportion of the LHC is located in the larger grana of the shade-acclimated chloroplast. Sun-acclimated leaves contain more xanthophyll carotenoids relative to chlorophyll (Lambers et al., 2008). Although shade-acclimated leaves may possess a higher light harvesting capacity and even higher chlorophyll content than sun-acclimated leaves, they possess lower photosynthetic capacity and are thus more susceptible to photodamage (Evans, 1989; Murchie et al., 2005).

In this study, the role of NPQ in the light response of photosynthesis in different leaf color cultivars is investigated. Using moderate and high light treatments, we measured photosynthetic characteristics, components of NPQ and ultrastructure of the chloroplasts in two rice genotypes: Zhefu802 (high chlorophyll) and Chl-8 (low chlorophyll). This study provides insights into the mechanism by which NPQ regulates photosynthetic processes, and gives insight for breeding cultivars with high photosynthetic efficiency.

## Materials and method

### Plant materials

Two rice genotypes, Zhefu802, a recurrent parent, and its near-isogenic line Chl-8 were selected for this experiment. Zhefu802 is a dark green leaf cultivar with a chlorophyll content that closely matches the mean chlorophyll content of widely used cultivars, while Chl-8 is a pale green leaf cultivar. Pre-germinated seeds of each cultivar were sown in 5 L buckets filled with 3 kg of soil. Two grams of compound fertilizer were then applied in each bucket when the plants had reached the 4-leaf stage. The seedlings were grown under natural conditions. Four days prior to experimentation, the plants were transferred to a growth cabinet with a 12 h day/night (d/n) cycle at 75/80% (d/n) relative humidity, 30/23°C (d/n) temperatures and 600 μmol m^−2^ s^−1^ light intensity. The experiments were conducted in the growth cabinet and commenced when the plants reached the 6-leaf stage. The plants were then subjected to either a moderate or high light treatment, including 600 μmol m^−2^ s^−1^ (moderate light) or 1200 μmol m^−2^ s^−1^ (high light) for 6 days. The youngest fully expanded leaves (seventh leaves) of the seedlings, which developed during light treatment, were randomly selected for chlorophyll fluorescence, gas exchange, and subsequent measurements. The design was completely randomized within the growth chamber with three replicates per treatment, and the entire experiment was performed in duplicate.

### Gas exchange measurement

The net photosynthetic rate (Pn), photorespiratory rate (Pr), and light response curve were determined using an infrared gas analyzer-based portable photosynthesis system (LI-6400; Li-Cor, Lincoln, NE, USA) mounted with a red/blue LED light source (6400-02B; Li-Cor) on the youngest fully developed leaves. Pr is defined as the difference between the Pn measured under low (2% O_2_) and ambient O_2_ concentration (Zelitch, 1992; Li, 2011). All measurements were conducted at 30°C, CO_2_ concentration of 400 μL L^−1^ and an ambient humidity of 75 ± 5%. Photosynthetic photon flux density (PPFD) at the leaf surface was controlled at 1000 μmol m^−2^ s^−1^ during Pn and Pr measurements.

### Measurements of Rubisco activity

The initial activity of Rubisco was measured according to the method described by Li et al. (2013). The youngest fully expanded leaves were snap-frozen in liquid nitrogen and preserved at −80°C until further use. Frozen leaf samples (0.3 g) were homogenized using a chilled mortar and pestle with 4 mL cooled extraction containing 50 mM Tris-HCl (pH 7.5), 10 mM MgCl_2_, 1 mM EDTA-Na_2_, 12.5% (v/v) glycerine, 10 mM β-mercaptoethanol and 1% PVP-40. The homogenate was centrifuged at 10000 × g for 20 min at 4°C. Rubisco activity was measured via coupling 3-phosphoglyceric acid formation with NADH oxidation. Rubisco activity measurements were taken in a 0.9 mL reaction mixture containing 55.56 mM HEPES–NaOH (pH 7.5), 11.11 mM NaHCO_3_, 20.22 mM MgCl_2_, 2.78 mM dithiothreitol, 1.11 mM EDTA-Na_2_, 11.11 U creatine phosphokinase, 11.11 U 3-phosphoglyceric phosphokinase, 1.11 U glyceraldehyde 3-phosphate dehydrogenase, 5.56 mM ATP, 0.17 mM NADH, 5.56 mM phosphocreatine, 0.67 mM ribulose diphosphate, 11.11 mM Tris-HCl (pH 7.5) and 0.1 mL leaf extract. The change in absorbance at 340 nm was monitored for 90 s.

### ATPase determination

The ruptured chloroplast suspension was prepared according to Li (1987). Ca^2+^-ATPase and Mg^2+^-ATPase were assayed in a 1-mL reaction solution containing 0.1 M Tris-HCl (pH 7.5), 50 mM NaCl, 5 mM ATP, 5 mM MgCl_2_ (2 mM CaCl_2_ for Ca^2+^-ATPase), 5 mM dithiothreitol and 2 μM EDTA, and 0.1 mL chloroplast suspension was added to the reaction solution. The reaction mixture was incubated for 10 min at 37°C, after which 0.1 mL 20% trichloroacetic acid was added to halt the reaction. Following centrifugation, the supernatant solution was analyzed for phosphorus content (Douce et al., 1973; Li, 1987).

### Chlorophyll fluorescence analysis

Chlorophyll fluorescence parameters were measured using a PAM-2500 chlorophyll fluorometer (Walz Heinz GmbH, Effeltrich, Germany). The plants were dark-adapted for 30 min prior to determination. The minimum fluorescence (F_o_) at open PSII centers was determined by measuring light, while the maximum fluorescence (F_m_) at closed PSII centers was examined after an application of a 0.8 s pulse of saturating light (6000 μmol m^−2^s^−1^) after 30 min in darkness. Maximum quantum efficiency of PSII (F_v_/F_m_) was defined as (F_m_−F_o_)/F_m_. Actinic light (red light) was applied to measure the steady-state chlorophyll fluorescence (F_s_). In the light-adapted state, ${\text{F}}_{\text{m}}^{\prime }$ was measured by applying a saturating pulse, while ${\text{F}}_{\text{o}}^{\prime }$ was measured by switching off the actinic light for 2 s after the saturating pulse and applying far-red light. NPQ was defined as F_m_/${\text{F}}_{\text{m}}^{\prime }$−1; actual quantum efficiency of PSII (Φ_PSII_) was defined as (${\text{F}}_{\text{m}}^{\prime }$−F_s_)/${\text{F}}_{\text{m}}^{\prime }$; photochemical quenching (qP) was defined as 1−(F_s_−${\text{F}}_{\text{o}}^{\prime }$)/(${\text{F}}_{\text{m}}^{\prime }$−${\text{F}}_{\text{o}}^{\prime }$) (Maxwell and Johnson, 2000; Baker, 2008).

### Determination of the different NPQ components and calculation of qE and qI

In high-light illumination, qT quenching is expected to be suppressed. Typical quenching analysis followed by relaxation analysis was applied to the dark-adapted, youngest fully developed leaves using actinic light of 1200 μmol m^−2^ s^−1^. ${\text{F}}_{\text{m}}^{\prime \prime }$ was defined as the maximal fluorescence during dark recovery after previous illumination. ${\text{F}}_{\text{m}}^{\prime }$ and ${\text{F}}_{\text{m}}^{\prime \prime }$ were determined after 10 min of illumination and after 10 min of dark recovery, respectively qE = F_m_/${\text{F}}_{\text{m}}^{\prime }$ − F_m_/${\text{F}}_{\text{m}}^{\prime \prime }$; qI = F_m_/${\text{F}}_{\text{m}}^{\prime \prime }$ − 1 (Krause and Jahns, 2003).

### Determination of malondialdehyde (MDA) content

Leaf samples (0.2 g) were ground in liquid nitrogen using a pestle and a mortar into which 5 mL ice-cold 10% (w/v) trichloroacetic acid had been added. The concentration of MDA was measured following the method of Dionisio-Sese and Tobita (1998). Briefly, the homogenate was centrifuged for 15 min at 10000 × g and 1.5 mL of supernatant was added to the same volume of a 0.67% (w/v) thiobarbituric acid solution containing 10% (w/v) trichloroacetic acid. The mixture was heated at 100°C for 30 min and the reaction was rapidly halted by applying the mixture to an ice bath. The cooled reaction solution was then centrifuged at 10000 × g for 10 min, and the absorbance of the supernatant was measured at 450, 532, and 600 nm. The MDA concentration was measured using (μM) = 6.45 × (OD532−OD600) − 0.56 × OD450.

### Hydrogen peroxide determination

The hydrogen peroxide content was extracted with 3-amino-1,2,4-triazole, after which the titanium–peroxide complex was measured following the method of Brennan and Frenkel (1977). Plant material (0.3 g) was homogenized in 4 mL of 10 mM 3-amino-1,2,4-triazole. After centrifugation for 25 min at 6000 × g, 0.1% titanium tetrachloride in 20% H_2_SO_4_ of 1 mL was added to 2 mL supernatant. The reaction solution was further centrifuged to remove the undissolved materials, and absorbance was recorded at 410 nm against a blank. The H_2_O_2_ concentration was examined using a standard curve plotted with a known concentration of H_2_O_2_.

### Superoxide anion (O^2−^) measurement

The rate of superoxide production was determined according to the method of Chaitanya and Naithani (1994). The frozen leaf samples (0.3 g) were homogenized in 100 mM sodium–phosphate buffer (pH 7.2) containing 1 mM diethyl dithiocarbamate, which can inhibit superoxide dismutase (SOD) activity. After centrifugation at 13000 × g for 20 min, the supernatant was used to measure the rate of superoxide anion (O^2−^) by its capacity to reduce nitroblue tetrazolium. The assay mixture (3 mL) contained the supernatant, 100 mM sodium phosphate buffer (pH 7.2), 1 mM diethyl thiocarbamate and 0.25 mM nitroblue tetrazolium. The absorbance was measured at 540 nm using a spectrophotometer (Lambda 25, Perkin Elamer, USA).

### Antioxidant enzyme activities determination

The youngest fully expanded leaves were sampled, immersed in liquid nitrogen and then stored at −80°C. Samples measuring 0.2 g were homogenized in 5 mL extraction buffer (100 mM sodium phosphate buffer, pH 7.0). The homogenates were centrifuged for 15 min at 10000 × g at 4°C, and then the supernatant solution was used for SOD, peroxidase (POD), ascorbate peroxidase (APX), and catalase (CAT) activity determinations. The SOD activity assay was based on the inhibition of the photoreduction of nitroblue tetrazolium as described by Giannopolitis and Ries (1977). The POD activity assay was based on the conversion of guaiacol to tetraguaiacol, which was monitored at 470 nm as described by Maehly and Chance (1954). The activity of CAT was measured according to the method of Aebi (1984). APX activity was measured in 100 mM sodium phosphate buffer (pH 7.0) of 2.9 mL, with 0.1 mM EDTA-Na_2_, 0.3 mM ascorbic acid and 0.06 mM H_2_O_2_. The reaction was initiated with the addition of 100 μL plant extract and the decreasing absorbance was measured at 290 nm for 90 s. One unit of APX was defined as the activity that could oxidize one micromole of ascorbic acid per minute at 25°C at pH 7.0 (Bonnecarrère et al., 2011).

### Ascorbate (ASA) and dithiothreitol (DTT) feeding

DTT is an inhibitor of violaxanthin de-epoxidase that promotes the induction of NPQ, whereas ASA is the cofactor for violaxanthin de-epoxidase. Even though ASA and DTT have diverse physiological roles in plant tissues, they have respectively been used as NPQ promoters and NPQ inhibitors to investigate the role of NPQ in plants, as they obviously contribute to changes in NPQ (Bilger and Björkman, 1990; Neubauer, 1993; Mohanty and Yamamoto, 1996; Xu et al., 1999; Ivanov and Edwards, 2000; Yin et al., 2010; Roháček et al., 2014; Naranjo et al., 2015; Quaas et al., 2015). The leaves of the rice plants grown under 600 μmol m^−2^s^−1^ were sprayed with 10 mM DTT in 0.005% (v/v) Tween-80 (DTT treatment), 15 mM ASA in 0.005% (v/v) Tween-80 (ASA treatment), and 0.005% (v/v) Tween-80 in water (control treatment). Gas exchange and chlorophyll fluorescence were determined 1 day following this treatment.

### Pigment measurements

Carotenoids were extracted from 0.2 g frozen leaf samples in pure acetone and separated by HPLC (HP1100, Agilent, Palo Alto, CA, USA) using a LiChrospher C18 column (Hypersil ODS 4.6 × 250 mm, 5 μm) as described by Havaux et al. (2005). Chlorophyll was extracted according to the method described in Sartory and Grobbelaar (1984). Briefly, 3 cm^2^ of fresh leaf samples were immersed in 20 mL 95% alcohol for 2 days. Chlorophyll a and b concentration was measured using a spectrophotometer, and the following calculations were used: chlorophyll a (C_a_ μg mL^−1^) = 13.95 (A_665_) − 6.88 (A_649_), chlorophyll b (C_b_ μg mL^−1^) = 24.96(A_649_) − 7.32 (A_665_).

### Quantitative real-time PCR analysis

Leaves were frozen in liquid nitrogen and stored at −80°C. Total RNA was extracted from 100 mg leaves using TRIpure reagent (Aidlab Biotechnologies, Beijing, China). RNA was converted into first-strand cDNA using the ReverTra Ace qPCR RT Master Mix (TOYOBO, Shanghai, China) with oligo (dT) as a primer. The resultant cDNA was used as a template for quantitative PCR amplification in a Thermal Cycler Dice Real Time System II (TaKaRa Biotechnology, Dalian, China) using SYBR Green I (TOYOBO) as a fluorescent reporter. Primers were designed to generate 150–250 base pair (bp) fragments using PRIMER5 software (Rozen and Skaletsky, 2000). Primers for qRT-PCR are listed in Supplementary Table 1. PCR and detection were performed as described previously (Feng et al., 2013). The 2^−ΔΔCT^ method was used to analyze the relative transcript levels in gene expression with the means from three replications.

### Transmission electron microscopy

Leaves were sampled within 1 h from the start of the light period. The segments of leaf were fixed at 4°C in 2.5% glutaraldehyde, and then treated with 1% osmium tetroxide overnight at 4°C. The fixed segments were dehydrated in a graded acetone series and embedded in Spurr's resin (Ladd). Transmission electron microscopy of chloroplast ultrastructure was performed using 40 nm ultrathin sections cut with a diamond knife on the ultramicrotome (Leica Ultracut R) and stained with uranyl acetate and lead citrate double staining. Chloroplasts were viewed under an electron microscope and electron micrographs were taken with a digital camera.

### Statistical analysis

Data were processed with SPSS 11.5. Tukey's least significant difference (LSD) at a significance level of 5% was used to compare the differences between treatments and between genotypes.

## Results

### Chlorophyll content and photosynthetic light-response curves

**Figure 1:**
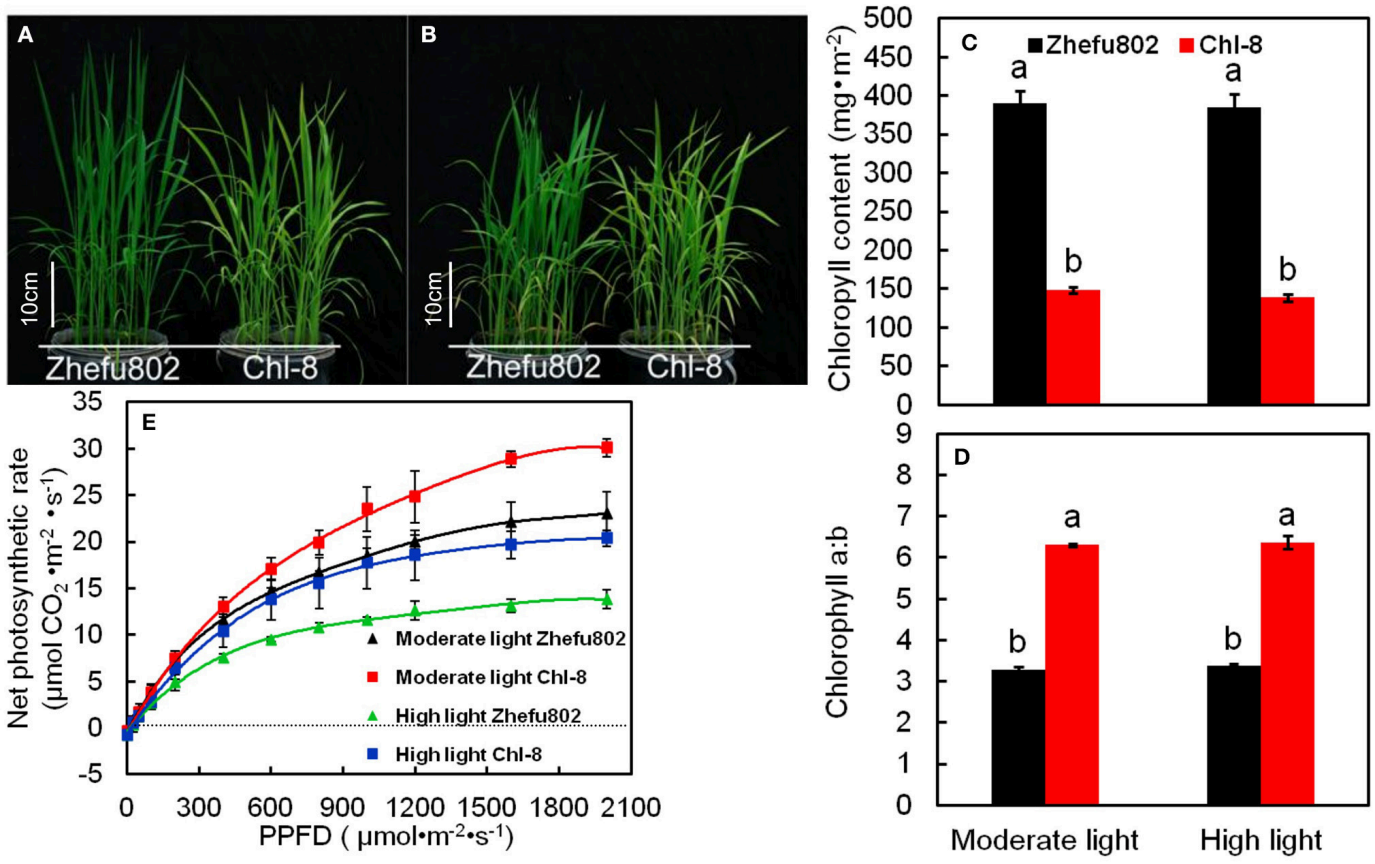
**Response of chlorophyll content and photosynthesis in the leaves of rice in different light intensity treatments. (A,B)** Plants were subjected to light intensities of 600 μmol m^−2^ s^−1^, moderate light **(A)** or 1200 μmol m^−2^ s^−1^, high light **(B)**. **(C–E)** chlorophyll content **(C)**, chlorophyll a:b ratio **(D)** and photosynthetic response curves **(E)** of rice leaves in each light treatment. PPFD, photosynthetic photon flux density. Vertical bars represent standard deviations (*SD, n* = 6). Different letters denote significant differences between moderate light and high light intensities in each rice genotype (*p* < 0.05).

Figures 1A,B show the performance of Zhefu802 and Chl-8 under different light treatments. At both light intensities, the chlorophyll content (a+b) per unit leaf area of leaves in Zhefu802 was about three times higher than that of Chl-8, and the chlorophyll a:b ratios in Chl-8 were higher than that in Zhefu802. However, no obvious difference in the chlorophyll content and chlorophyll a:b ratio were observed between the two light treatments in both rice genotypes (Figures 1C,D). However, Chl-8 possessed higher Pn than Zhefu802 under both moderate and high light (Figure 1E). The maximum Pn of Chl-8 was higher than Zhefu802 by about 24% under moderate light, while under high light the maximum Pn of Chl-8 was about 47% higher than Zhefu802.

### CO_2_ assimilation and related enzyme activities

**Figure 2:**
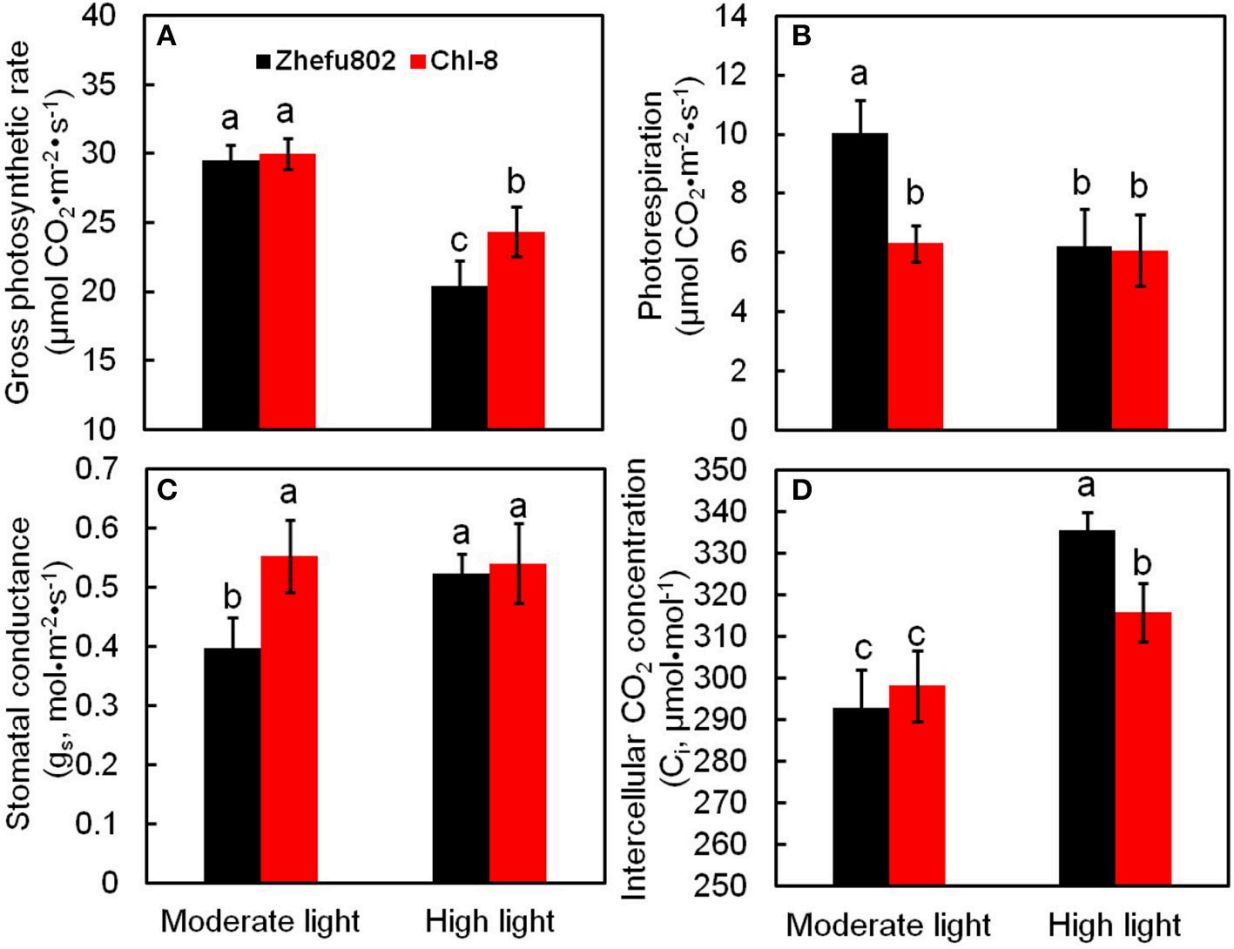
**Differences in photosynthetic characteristic and photorespiration of two rice genotypes (Chl-8 and Zhefu802) under different light intensities. (A)** Gross photosynthetic rate. **(B)** Photorespiration. **(C)** Stomatal conductance. **(D)** Intercellular CO_2_ concentration. Vertical bars represent *SD* (*n* = 6). Different letters denote significant differences between plants in the moderate light and high light intensities in both rice genotypes (*p* < 0.05).

Under moderate light, the differences between the genotypes in Pn might primarily be the result of differences in Pr, because no significant difference in gross photosynthesis (Pn+Pr) was observed between the two genotypes, whereas the Pr in Zhefu802 was significantly higher than in Chl-8. There was no significant difference in Pr between the two genotypes under high light, but gross photosynthesis in Chl-8 was significantly higher than in Zhefu802 (Figures 2A,B). Stomatal conductance in Chl-8 was significantly higher than in Zhefu802 under moderate light intensity, whereas no significant difference was observed in the intercellular CO_2_ concentration (Figures 2C,D). This suggested that stomatal limitation was not responsible for the lower Pn observed in Zhefu802 under moderate light. Under high light conditions, the intercellular CO_2_ concentrations were significantly increased in both genotypes, but a greater increase was evident in Zhe802. This indicated that non-stomatal limitation in Pn occurred under high light, particularly for Zhefu802.

**Figure 3:**
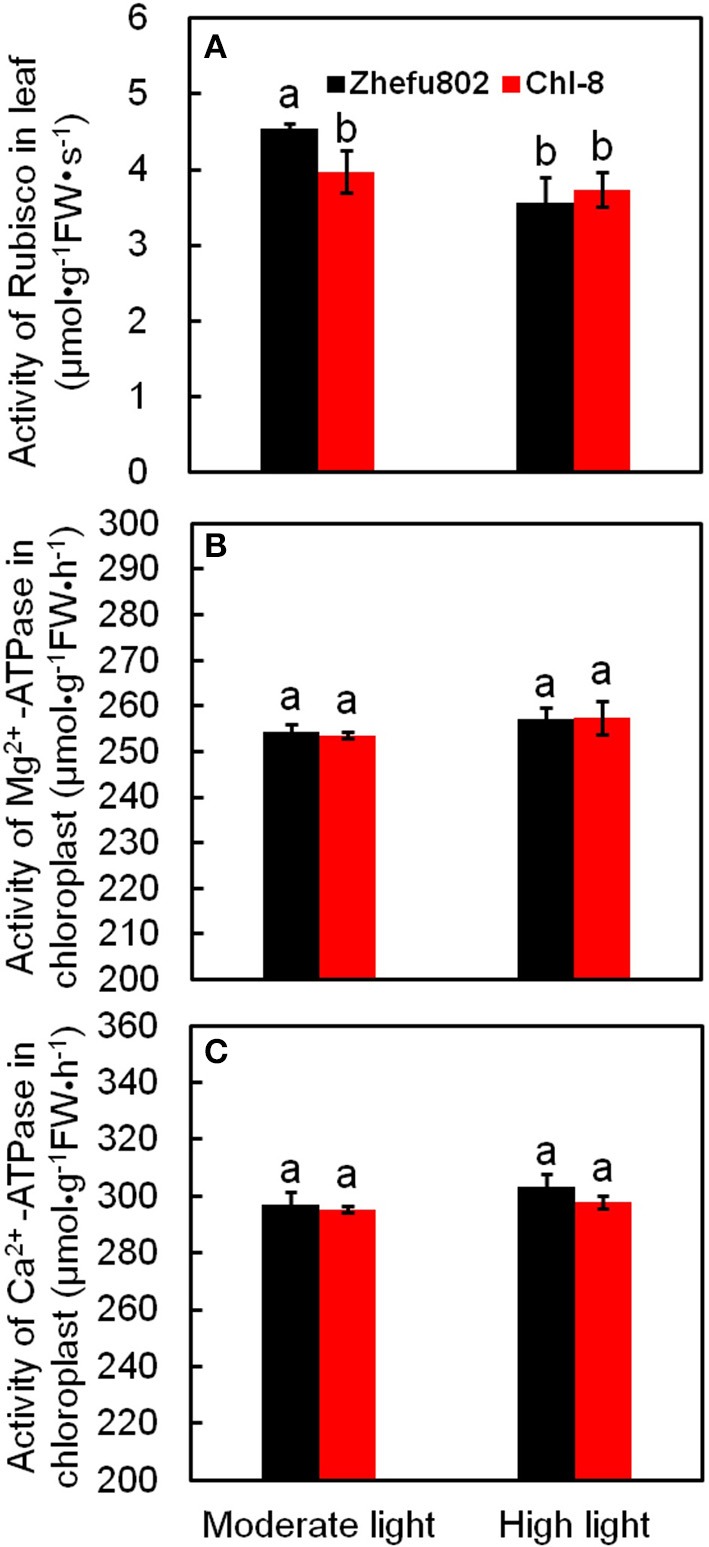
**Differences in enzyme activities involved in CO_**2**_ assimilation of two rice genotypes (Chl-8 and Zhefu802) under different light intensities. (A)** Rubisco activity. **(B)** Mg^2+^-ATPase activity. **(C)** Ca^2+^-ATPase activity. Vertical bars represent *SD* (*n* = 6). Different letters denote significant differences between plants in the moderate light and high light intensities in both rice genotypes (*p* < 0.05).

Rubisco is a key enzyme involved in the Calvin cycle. In the chloroplast, ATPase catalyzes the formation of ATP to provide energy for CO_2_ assimilation. However, light intensity influences the synthesis and decomposition of ATP. ATPase is activated by Mg^2+^ and Ca^2+^, thus ATPases can be divided into two types: Mg^2+^-ATPase and Ca^2+^-ATPase. There was no significant difference in Mg^2+^-ATPase and Ca^2+^-ATPase between the two genotypes and between the light treatments (Figure 3). The initial activity of Rubisco in Chl-8 was significantly higher than in Zhefu802 under moderate light, whereas no significant difference was observed under high light (Figure 3A).

### Chlorophyll fluorescence analysis

**Figure 4:**
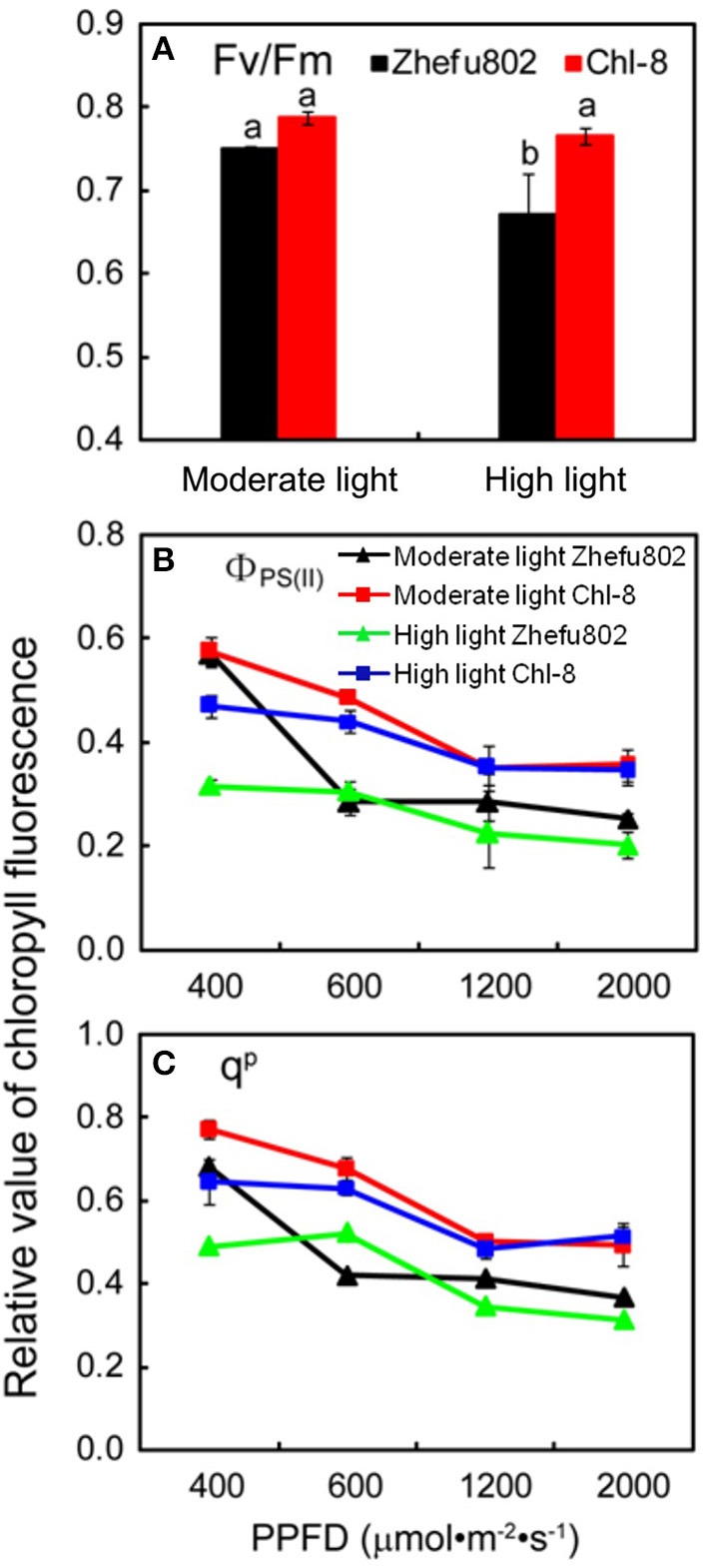
**Chlorophyll flourescence analysis. (A)** Maximum quantum efficiency of PSII, Fv/Fm; **(B)** Actual quantum efficiency of PSII, Φ_PS(II)_; **(C)** Photochemical quenching, q^P^. PPFD, Photosynthetic photon flux density. Vertical bars represent *SD* (*n* = 6). Different letters denote significant differences between plants in the moderate light and high light treatments in both rice genotypes (*p* < 0.05).

Chlorophyll fluorescence analysis via PAM-2500 mainly reflects the activity of PS II. There were no significant differences in F_v_/F_m_ between Zhefu802 and Chl-8 under moderate light (Figure 4A). In contrast, a significantly lower F_v_/F_m_ was observed in the leaves of Zhefu802 compared with Chl-8 under high light conditions. Irrespective of the actinic light densities, the Φ_PSII_ and qP of leaves in Chl-8 were always higher than those of Zhefu802 (Figures 4B,C). In both genotypes and under both light treatments, the Φ_PSII_ and qP measurements were reduced as the measured actinic light densities increased.

### Non-photochemical quenching

**Figure 5:**
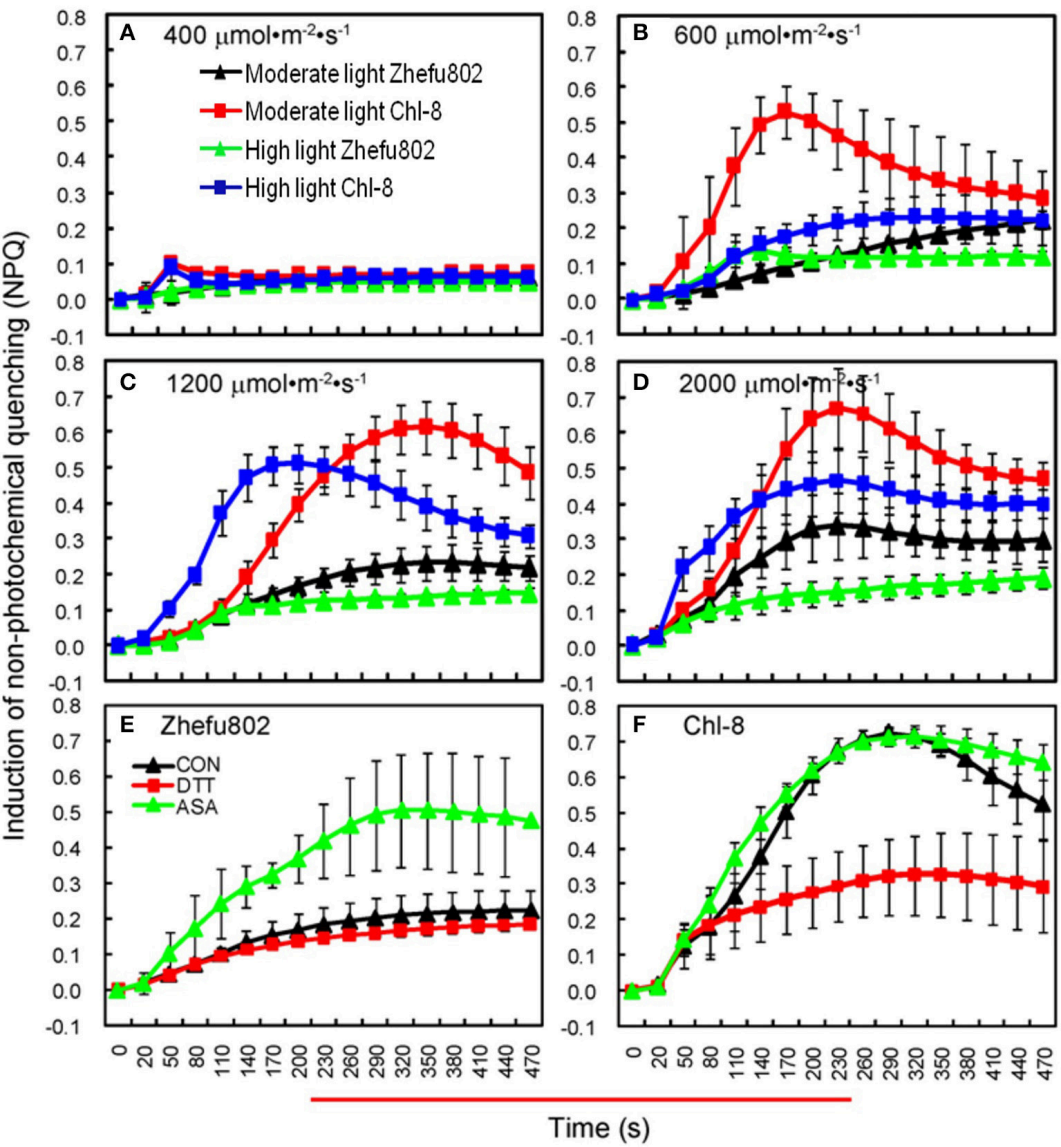
**Induction of NPQ under different light intensities. (A–D)** NPQ under light intensities of 400 **(A)**, 600 **(B)**, 1200 **(C)**, and 2000 μmol m^−2^ s^−1^ **(D)**. **(E,F)** The induction of NPQ in rice plants sprayed with dithiothreitol and ascorbic acid in the 1200 μmol m^−2^ s^−1^ light treatment in Zhefu802 **(E)** and Chl-8 **(F)**. Vertical bars denote *SD* (*n* = 6).

A higher NPQ was observed in Chl-8 compared with Zhefu802 under both light treatments (Figure 5). For both rice genotypes grown under moderate and high light intensities, the NPQ was very low when subjected to actinic light densities of 400 μmol m^−2^ s^−1^ (Figure 5A), but was higher at light densities above 600 μmol m^−2^ s^−1^ (Figures 5B–D). The highest NPQ was observed at the actinic light density of 2000 μmol m^−2^ s^−1^. However, this was only slightly higher than that determined at 1200 μmol m^−2^ s^−1^, particularly in Chl-8. Nevertheless, the NPQ of leaves in Chl-8 was always higher than Zhefu802, irrespective of the light intensities imposed.

### Components of non-photochemical quenching

**Figure 6:**
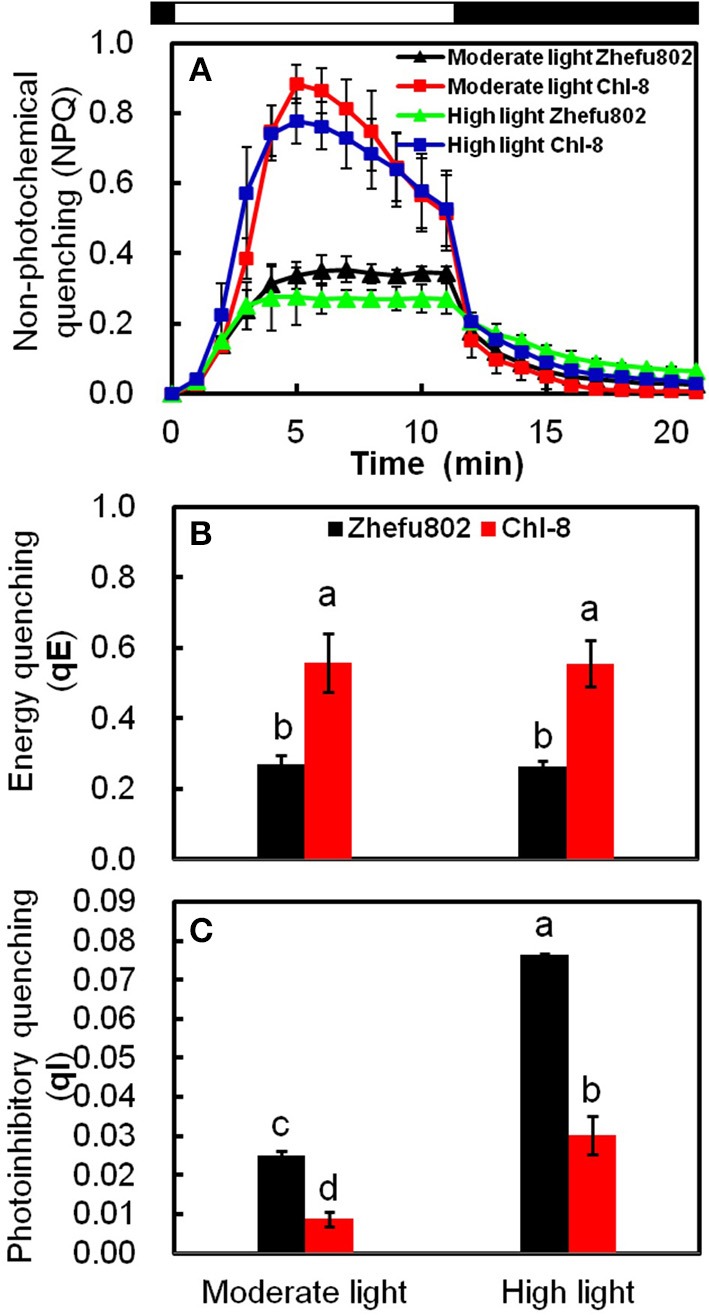
**NPQ relaxation analysis and calculation of energy quenching (qE) and photoinhibitory quenching (qI). (A)** Time-course of NPQ in 30 min dark-adapted leaf. Black bars represent dark, white bar represents actinic light of 1200 μmol m^−2^ s^−1^. qE **(B)** and qI **(C)** were calculated. Vertical bars represent *SD* (*n* = 6). Different letters denote significant differences between light treatments in each rice genotype (*P* < 0.05).

The determination of the different NPQ components was made possible by their different relaxation times during a period of darkness following high light illumination. Figure 6A depicts the time-course of NPQ under an actinic light density of 1200 μmol m^−2^ s^−1^. Figures 6B,C show the results of the dark relaxation kinetics from Figure 6A: the fast (qE) and slow relaxing (qI) component of NPQ. A large proportion of the NPQ was explained by qE. In contrast to Zhefu802, Chl-8 possessed a significantly higher qE and a lower qI under both moderate and high light treatments. There were significantly lower levels of qI in both genotypes under the moderate light treatment compared with the high light treatment.

### Reactive oxygen species (ROS) and scavengers

**Figure 7:**
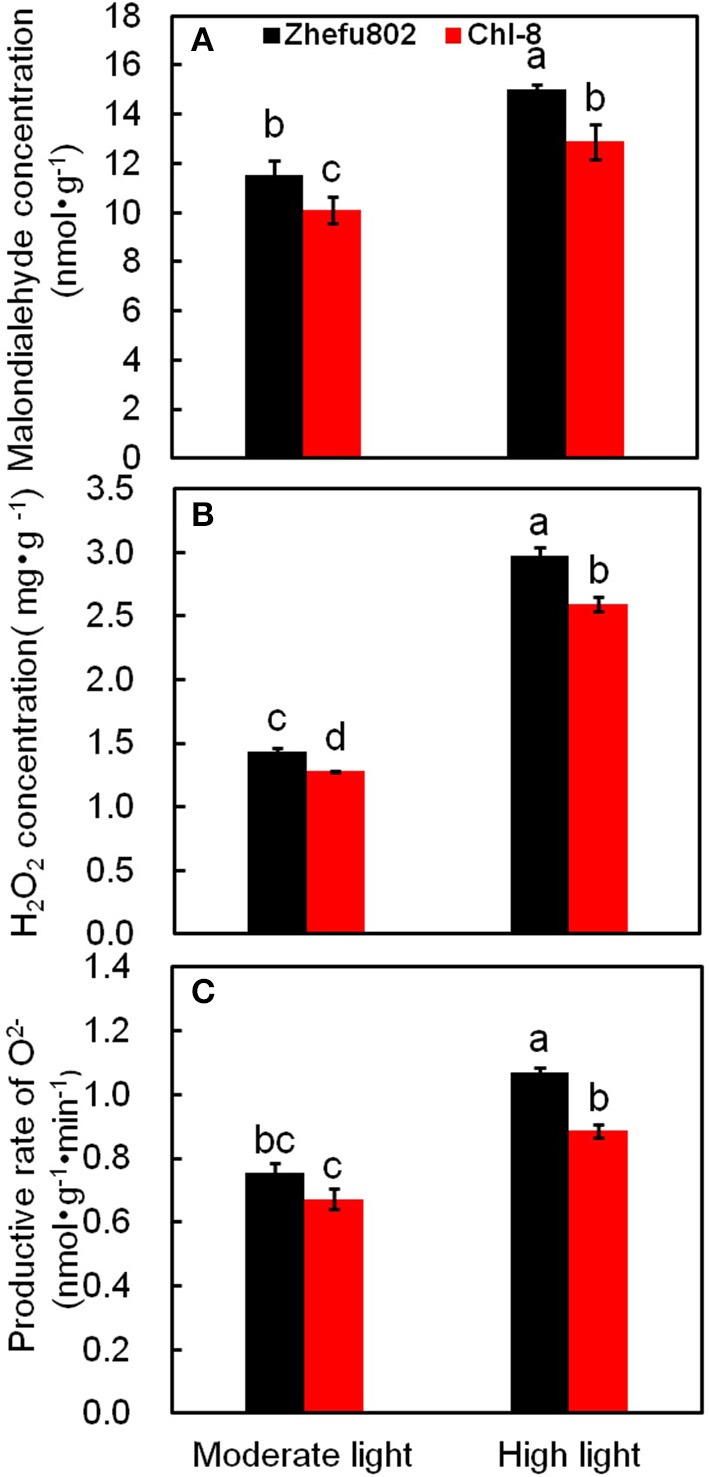
**Changes in the content of MDA (A), H_**2**_O_**2**_ (B), and O^**2−**^ (C) in rice leaves under different light intensities**. Vertical bars represent *SD* (*n* = 6). Different letters denote significant differences between plants in the moderate light and high light treatments in both rice genotypes (*p* < 0.05).

Under moderate light, the concentrations of MDA and H_2_O_2_ as well as the productive rate of superoxide (O^2−^) were higher by ~18, 12, and 10% in Zhefu802 compared with Chl-8. Under high light, the difference observed between the genotypes was even greater: the levels of MDA, H_2_O_2_and superoxide in Zhefu802 were about 20, 21, and 19% higher than in Chl-8 (Figure 7).

**Figure 8:**
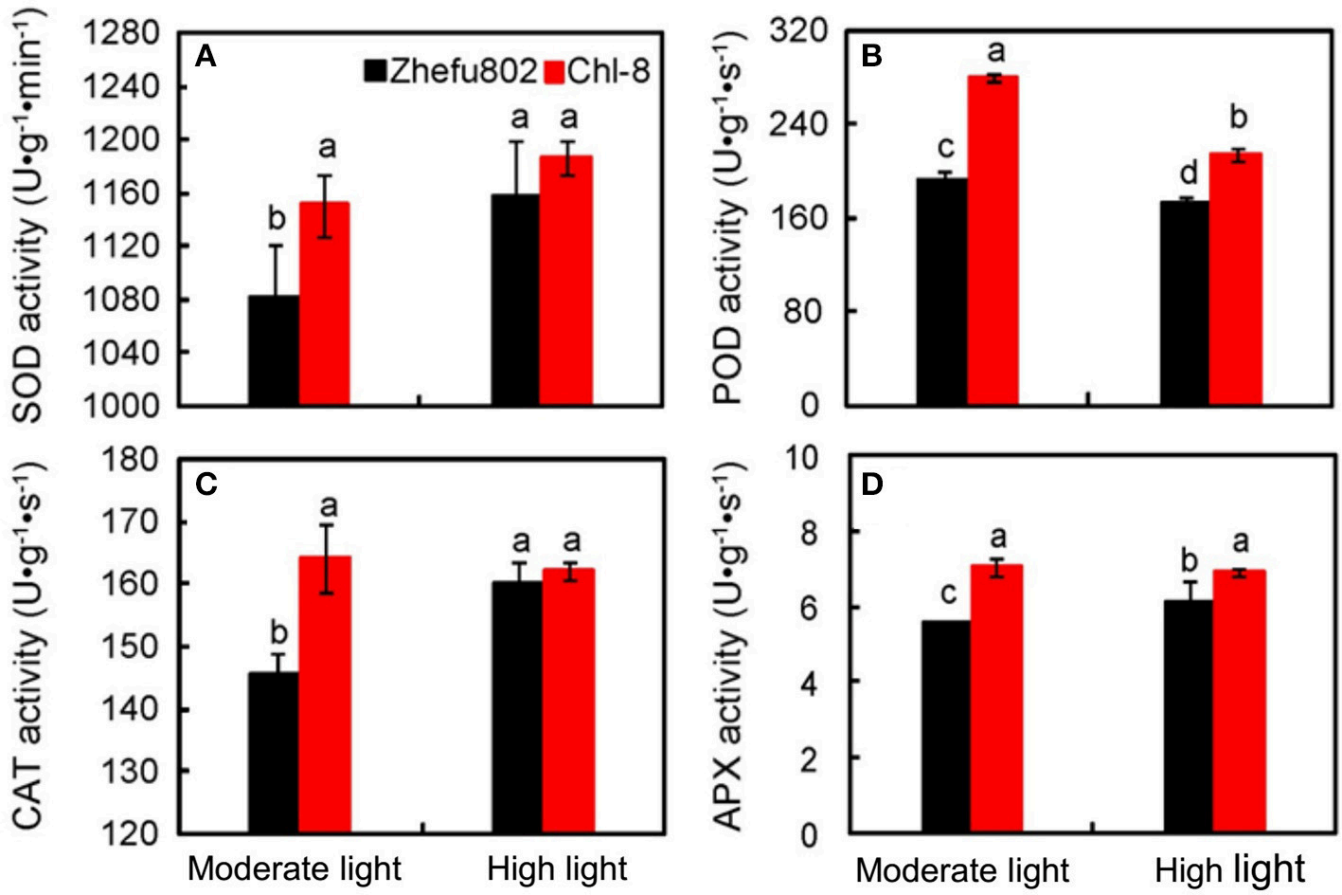
**Differences in antioxidant enzyme activities of SOD (A),** POD **(B)**, CAT **(C)**, and APX **(D)** in leaves of rice under different light intensities. Vertical bars represent *SD* (*n* = 6). Different letters denote significant differences between plants in the moderate light and high light treatments in both rice genotypes (*p* < 0.05).

The scavengers of ROS, including POD, CAT, SOD, and APX were significantly higher in Chl-8 than Zhefu802 under moderate light (Figure 8). However, the magnitude of the differences between genotypes was reduced under high light, and furthermore, there were no significant differences in the activities of SOD and CAT between Zhefu802 and Chl-8 under high light stress. Under low light stress, POD activity was significantly lower by about 10% in Zhefu802 and about 23% in Chl-8 compared with moderate light stress (Figure 8B).

### Effect of DTT and ASA on photosynthesis and chlorophyll fluorescence

To further confirm the effect of NPQ induction on rice plants, Pn, and chlorophyll fluorescence parameters of the leaves treated with DTT (NPQ inhibitor), ASA (NPQ promoter) or 0.005% (v/v) Tween-80 (control) were analyzed under moderate light (Table 1). The induction of NPQ was affected by foliage spraying with ASA and DTT (Figures 5E,F). In the ASA treatments, there was a significant increase in NPQ in Zhefu802 but only a slight increase was observed in Chl-8. In contrast, in the DTT treatments, there was a significant decrease in NPQ in Chl-8, but only a slight decrease in Zhefu802 which may be on account of Zhefu802 itself has very low NPQ. For both genotypes, their Pn and F_v_/F_m_ values were increased by treatment with ASA, while treatment with DTT resulted in a decrease in these values compared with control treatment. Similar results were also observed with respect to Φ_PSII_ and qP in Zhefu802 when sprayed with ASA and DTT respectively. Nevertheless, the highest Φ_PSII_ and qP were observed in Chl-8 in control treatment, followed by the ASA and DTT treatments.

**Table 1:** **Characteristics of the net photosynthetic rate and chlorophyll fluorescence parameters of rice leaves sprayed with DTT (NPQ inhibitor), ASA (NPQ promoter) or 0.005% (v/v) Tween-80 (control) in two rice genotypes (Chl-8 and Zhefu802) in the moderate light treatment**.

**Genotypes**	**Treatments**	**Pn**	***F_v_/F_m_***	**Φ_PSII_**	**qP**
	Control	17.73d	0.719c	0.334d	0.499bc
Zhefu802	DTT	15.96e	0.714c	0.303e	0.444c
	ASA	20.00c	0.744b	0.401c	0.603a
	Control	23.26b	0.788a	0.458a	0.642a
Chl-8	DTT	19.92c	0.750b	0.394c	0.526b
	ASA	24.55a	0.791a	0.420bc	0.604a

*Net photosynthetic rate and chlorophyll fluorescence parameters were determined on leaves attached to the plants 1 day after foliage was sprayed (n = 6). Different letters denote significant difference (P < 0.05) within the same rice genotype*.

### Xanthophyll cycle pigments

**Figure 9:**
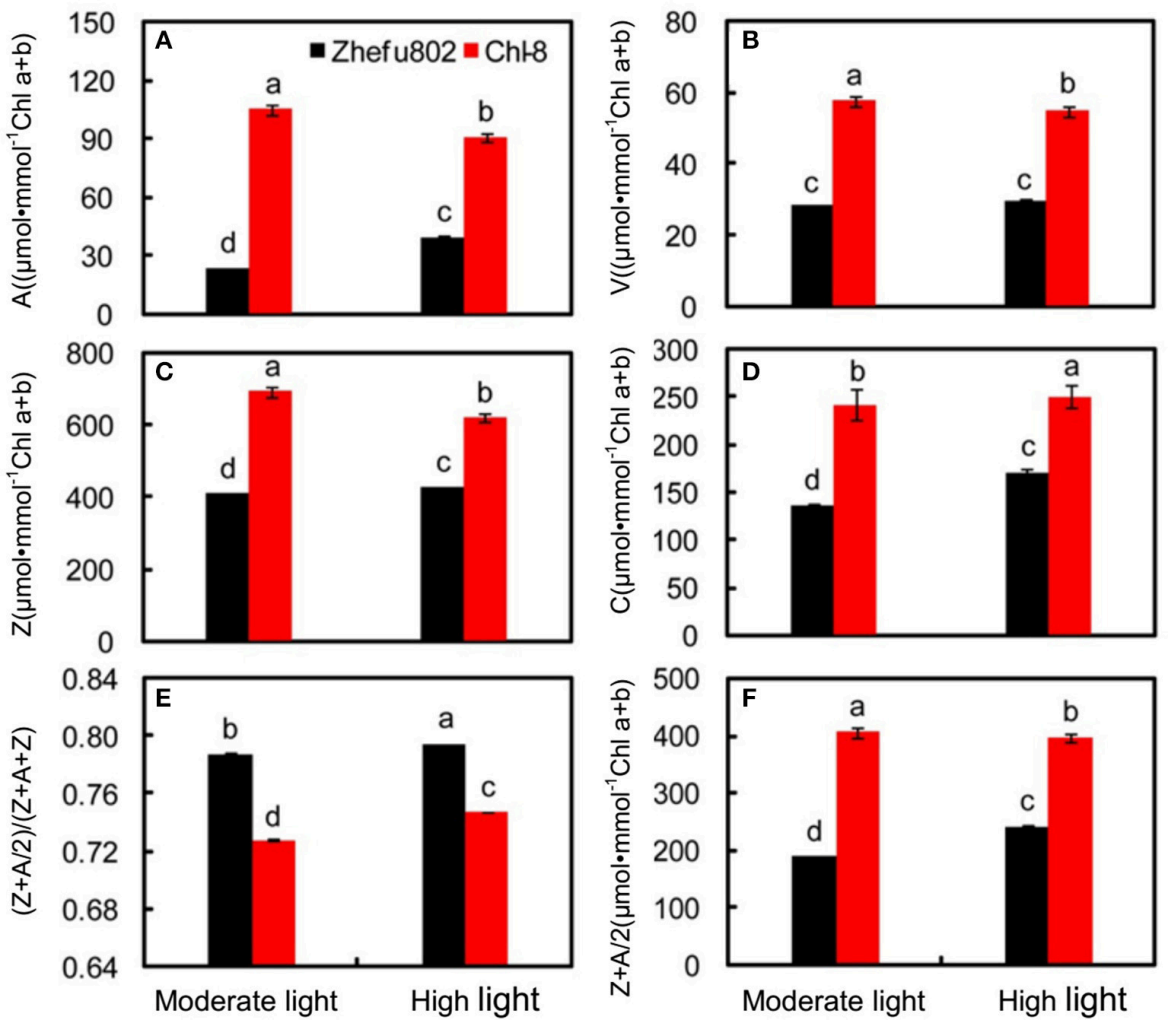
**Pigment characteristics of rice leaves under different light intensities. (A)** Antheraxanthin, A; **(B)** violaxanthin, V; **(C)** Zeaxanthin, Z; **(D)** carotenoids, C; **(E)** (Z+A/2)/(Z+A+V); **(F)** Z+A/2. Vertical bars represent *SD* (*n* = 6). Different letters denote significant differences between plants in the moderate light and high light treatments in both rice genotypes (*p* < 0.05).

Xanthophyll cycle pigments, including violaxanthin, antheraxanthin and zeaxanthin, and carotenoids were analyzed on the basis of chlorophyll. The xanthophyll cycle pool size was significantly higher in Chl-8 compared with Zhefu802 under both moderate and high light treatments (Figure 9). Zeaxanthin + antheraxanthin/2 contents in Chl-8 were significantly higher than in Zhefu802 (Figure 9F). High light treatments decreased antheraxanthin, zeaxanthin and violaxanthin levels in Chl-8, antheraxanthin and zeaxanthin levels in Zhefu802. But there was little difference in the violaxanthin content of Zhefu802 between the moderate and high light treatments (Figures 9A,C,D). Compared with moderate light, high light appears to have significantly increased the carotenoid content of leaves in both rice genotypes (Figure 9D). Xanthophyll cycle pigments calculated as (Z+A/2)/(V+A+Z) were significantly higher in Zhefu802 than Chl-8 under both moderate and high light treatments (Figure 9E).

### Quantitative real-time PCR analysis

**Figure 10:**
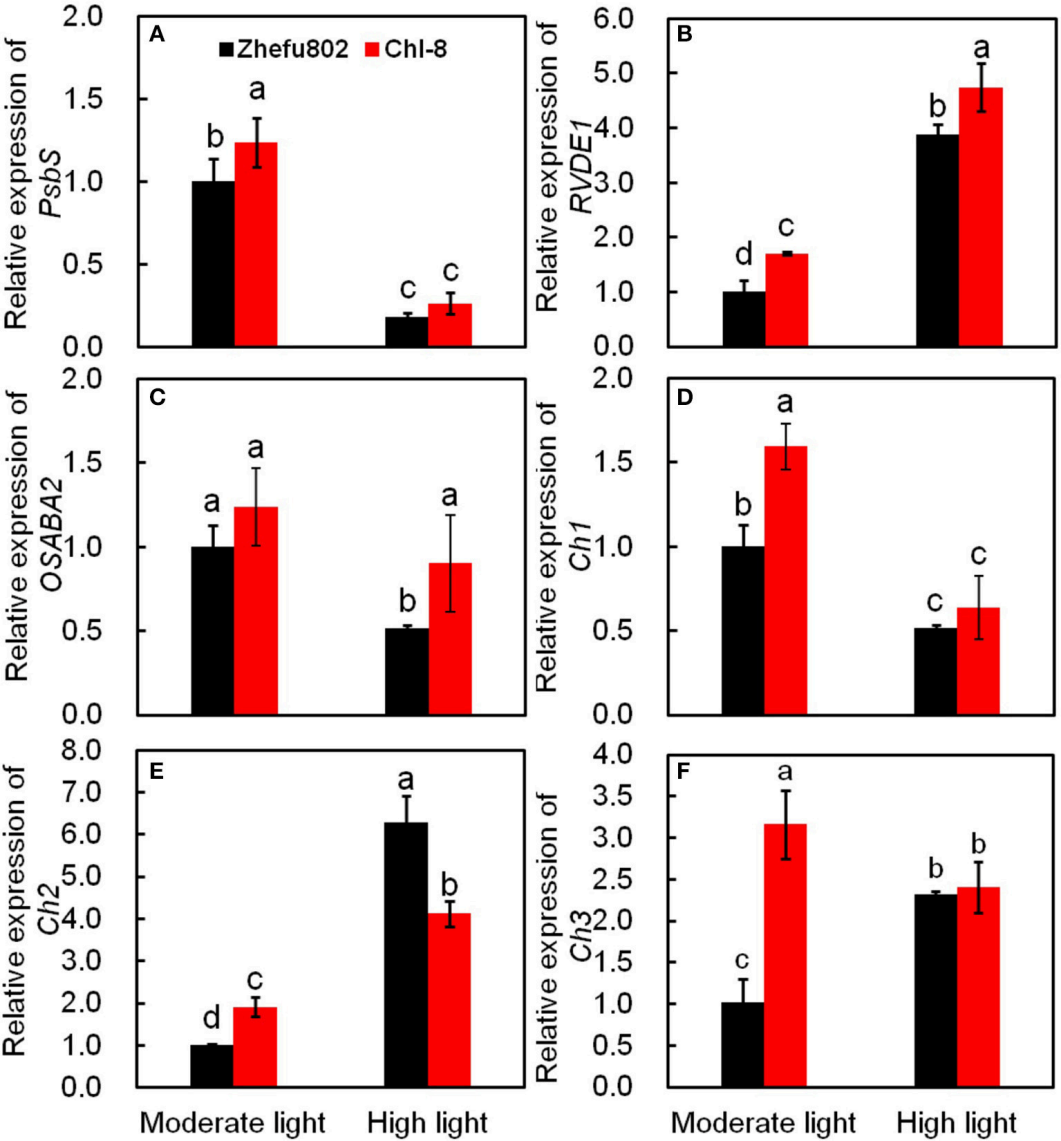
**Quantitative real-time PCR analysis**. Expression of *PsbS1* **(A)**, violaxanthin de-epoxidase (*RVDE1*, **B**), Zeaxanthin epoxidase (*OSABA2*, **C**), beta-carotene hydroxylase (*Ch1*, **D**), beta-carotene hydroxylase2 (*Ch2*, **E**) and beta-carotene hydroxylase3 (*Ch3*, **F**). Vertical bars represent *SD* (*n* = 6). Different letters denote significant differences between moderate light and high light in each rice genotype (*p* < 0.05).

Six genes related to the induction of NPQ were determined. Violaxanthin de-epoxidase (*RVDE1*) catalyzes the conversion of violaxanthin to zeaxanthin via the intermediate antheraxanthin, whereas zeaxanthin epoxidase (*OSABA2*) catalyzes the conversion of zeaxanthin to violaxanthin via antheraxanthin. β-carotene hydroxylase (*Chs*) is an enzyme catalyzing the conversion of β-carotene to zeaxanthin. Rice (*Oryza sativa* L.) has two *PsbS* genes, *PsbS1* and *PsbS2*. However, only inactivation of *PsbS1* results in plants deficient in qE (Zulfugarov et al., 2014). The relative expressions of all genes, with the exception of *OSABA2*, in Chl-8 were significantly higher than those in Zhefu802 under moderate light (Figure 10). *RVDE1, Ch2* were significantly higher in both genotypes under high light (Figures 10B,E). In contrast, high light reduced the relative expression of *PsbS1, Ch1*, and *OSABA2*, and a marked decrease in *OSABA2* was observed in Zhefu802, but not Chl-8 (Figures 10A,C,D). Under high light, a significantly higher relative expression of *RVDE1* was observed in Chl-8 than Zhefu802, while the expression of *Ch2* in Chl-8 was significantly lower than Zhefu802. Among these genes, the differences in the relative expression of *PsbS1, Ch1*, and *Ch3* between the genotypes were reduced when subjected to high light compared with those under moderate light conditions (Figures 10A,D,F).

### Chloroplast ultrastructure

**Figure 11:**
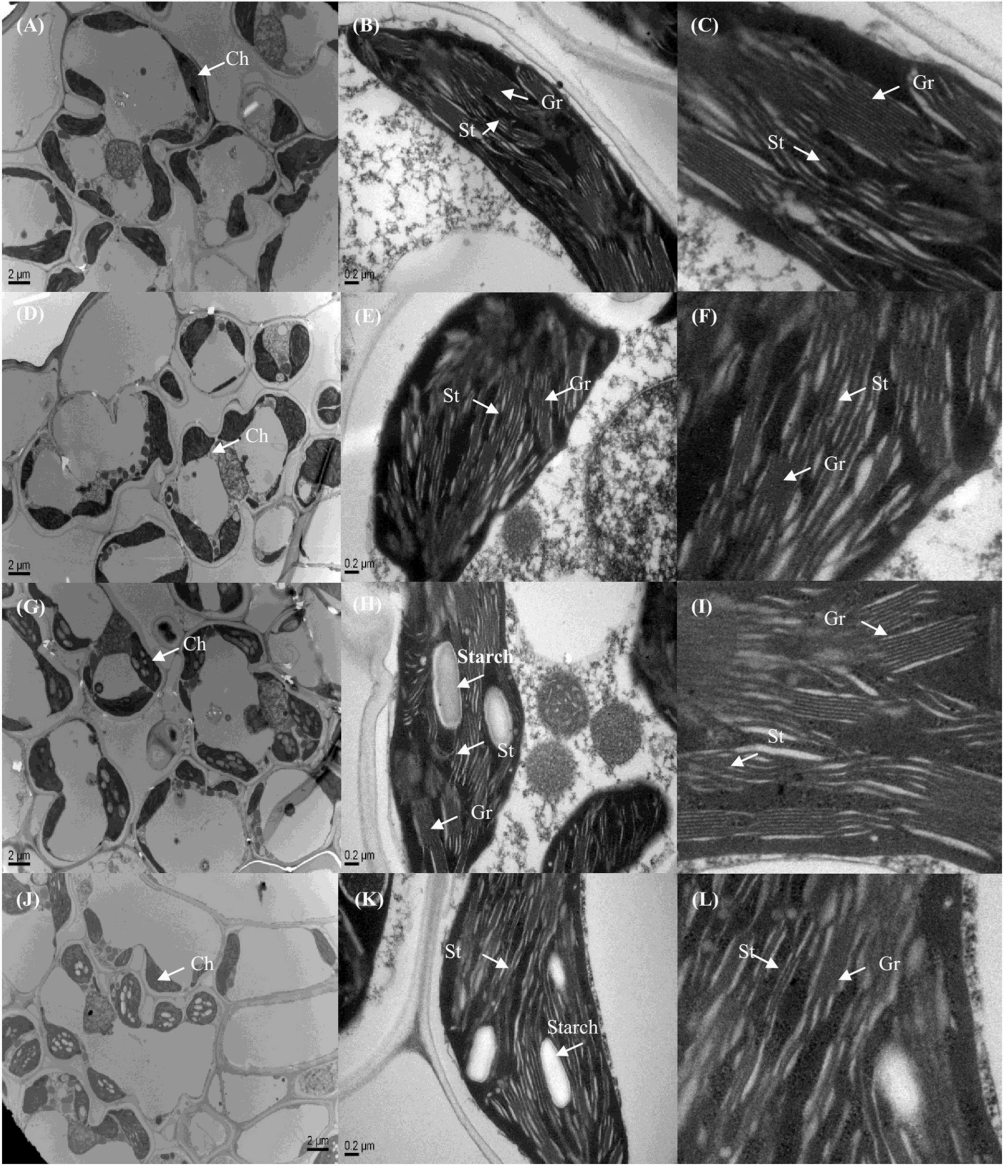
**Representative electron micrographs of chloroplasts in mesophyll cells of rice under different light intensities. (A–F)** Chloroplast ultrastructure of Zhefu802 **(A–C)** and Chl-8 **(D–F)** under 600 μmol m^−2^ s^−1^. **(G–L)** Chloroplast ultrastructure of Zhefu802 **(G–I)** and Chl-8 **(J–L)** under 1200 μmol m^−2^ s^−1^. **(C,F,I,L)** Magnification of the chloroplasts from **(B,E,H,K)** respectively Ch, chloroplast; Gr, granal thylakoid; St, stromal thylakoid; Starch, starch granule.

**Figure 12:**
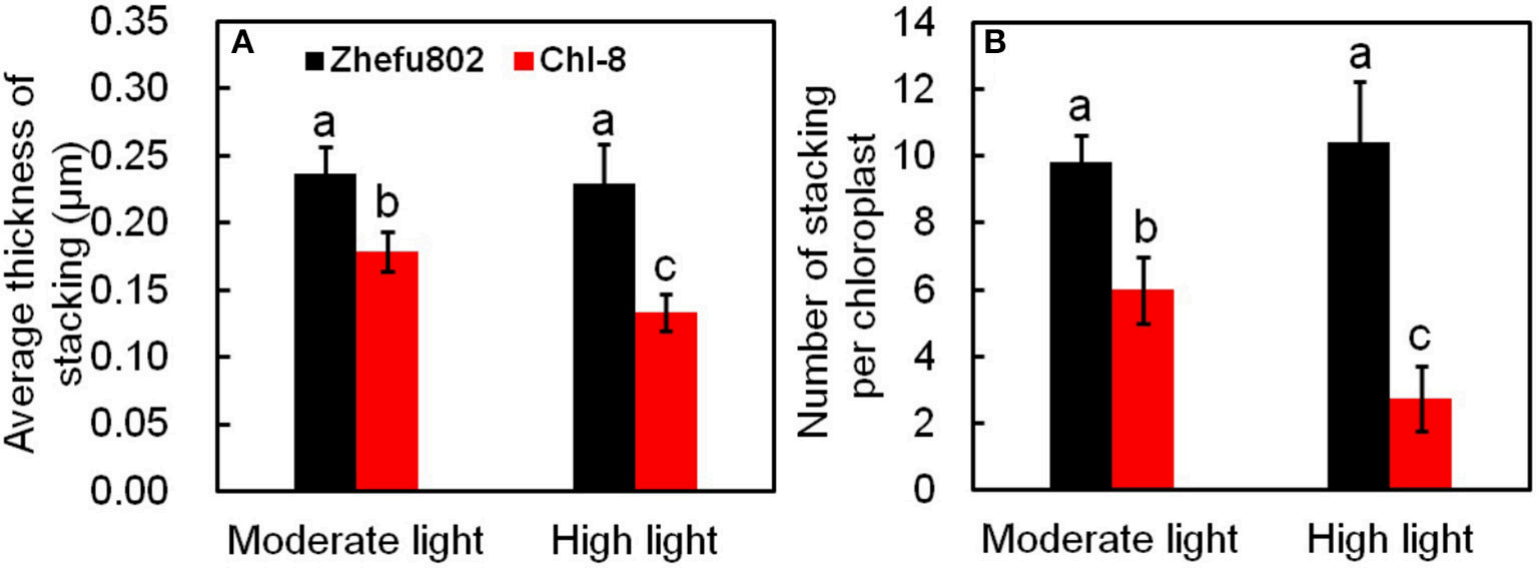
**Statistical analysis of electron micrographs of chloroplasts in mesophyll cells**. **(A)** Average thickness of stacking. **(B)** Number of stackings per chloroplast. Vertical bars represent *SD* (*n* = 6). Different letters denote significant differences between plants in the moderate light and high light treatments in both rice genotypes (*p* < 0.05).

On an ultrastructural level, changes also occurred in the chloroplasts. The thylakoid membranes in the chloroplasts of higher plants are differentiated into appressed and non-appressed membranes. Chl-8 possessed both thinner and fewer grana stacks compared with Zhefu802, irrespective of the light intensities imposed (Figures 11, 12). The number of stacks per chloroplast as well as the thickness of the stacks in Chl-8 under high light were significantly less than that under moderate light (Figure 12B). Starch granules accumulated in the chloroplast under high light treatment (Figures 11G–L), but there was no significant difference in the size of the chloroplast.

## Discussion

The LHC consists of chlorophyll a, chlorophyll b, and carotenoids, which primarily transfer absorbed light energy to the reaction center complexes of PSI and PSII, respectively. However, gas change measurements conducted under 1000 μmol m^−2^ s^−1^ and at 25°C in the field discovered low chlorophyll rice mutants with high photosynthetic rates (Wang et al., 2012). A similar pattern was also observed in our experiments: the Pn of Chl-8 was significantly higher than that of Zhefu802, even though the chlorophyll content of Chl-8 was about three times lower than Zhefu802. The value of chlorophyll ab ratio in Chl-8 was higher than that in Zhefu802 (Figure 1). These differences between the genotypes were correlated with differences in chloroplast ultrastructure. The larger proportion of the LHCs are located in the appressed thylakoid membrane (Lambers et al., 2008). The significantly thinner and fewer stacks observed in Chl-8 were associated with lower chlorophyll content observed (Figure 12). The chlorophyll a:b ratio is lower in shade-acclimated leaves than in sun-acclimated leaves. Shade-acclimated leaves contain relatively more chlorophyll in the LHCs; primarily chlorophyll b (Lichtenthaler and Babani, 2004). Although shade-acclimated leaves may have a higher light harvesting capacity than sun-acclimated leaves, they possess lower photosynthetic capacity and are thus more susceptible to photodamage (Murchie et al., 2005). Zhefu802 was discovered to be more susceptible to photodamage under high light than Chl-8.

Although greater Pn values were observed in Chl-8 than Zhefu802 under moderate light, there was no obvious difference in the gross photosynthetic rate (Pn+Pr) between the genotypes (Figure 2A). This phenomenon suggested that Pr resulted in a decrease in the Pn of Zhefu802 under moderate light. The difference in photorespiration might be related to plants differing in their NPQ induction abilities. A higher NPQ was observed in Chl-8 compared with Zhefu802 in both light treatments (Figures 5, 6). Jiang et al. (2006) reported that the combination of leaf angle, photorespiration, and thermal dissipation depending on the xanthophyll cycle could protect young leaves against excess light energy. This assumption was confirmed by the previous results of D'Ambrosio et al. (2006), who reported a higher NPQ in *Beta vulgaris* L. plants under 2% O_2_ compared with plants under 21% O_2_, whereas the photorespiration rate of the plants under 2% O_2_was lower than those under 21% O_2_, regardless of temperature. Additionally, under drought stress, a higher NPQ was observed in drought-resistant plants compared with drought-susceptible plants, while the photorespiration of drought-resistant plants was significantly lower than drought-susceptible plants (Beis and Patakas, 2012). Although photorespiration can dissipate excess reducing equivalents and energy to reduce the generation of ROS, it is also a source of H_2_O_2_; a redox signal in the regulation of redox homeostasis. Generally, photorespiration is proposed as an important photoprotective mechanism to mitigate oxidative stress (Kangasjärvil et al., 2011; Suzuki et al., 2012; Voss et al., 2013) Thus, an important synergy or complementation is likely to exist between NPQ and photorespiratory metabolism in order to maintain intracellular redox homeostasis. Some other photoprotective mechanisms, such as cyclic electron transport and water-water cycle, have been identified as closely associated with NPQ; even regulating the induction of NPQ (Johnson et al., 1994; Kramer et al., 2003; Miyake et al., 2005). However, the mechanisms underlying the interaction between NPQ and photorespiration remain elusive.

Under high light conditions, there was a marked increase in ROS including MDA and O^2−^, and particularly H_2_O_2_, in both rice genotypes compared with plants grown under moderate light(Figure 8). This result is in agreement with previous studies that have found the generation of high levels of ROS to be a contributing factor to the damage of organelles and protein complexes under high light conditions (Suzuki et al., 2012; Roach et al., 2015). ROS levels can rise significantly during periods of biotic or abiotic stress (Apel and Hirt, 2004). However, plants possess an extensive spectrum of antioxidants and antioxidative enzymes to manage high stress situations and ensure optimal cellular ROS concentrations to avoid damage, while allowing signaling to proceed. This defense and signaling system is highly dynamic and involves both the generation and scavenging of ROS to retain balanced levels in plant cells (Mittler et al., 2004, 2011). In our experiments, higher increases in ROS were discovered in Zhefu802 compared with Chl-8 under high light (Figure 7). Interestingly, Zhefu802 also possessed increased levels of the ROS scavenging enzymes such as SOD, APX, and CAT (Figure 8). A higher presence of ROS scavengers could be attributed to the higher production of ROS. Though ROS cause harmful oxidation, they are also powerful signaling molecules that are involved in priming acclamatory responses to stress stimuli (Jones and Dangl, 2006; Foyer and Noctor, 2009). In this case, higher antioxidant enzyme activities may have been induced to scavenge the higher levels of ROS.

In this study, few differences in the activities of Rubisco, Mg^2+^-ATPase, and Ca^2+^-ATPase were identified between the two genotypes (Figure 3), indicating their equal capacity for CO_2_ assimilation. No significant stomatal limitation was observed in both genotypes (Figures 2C,D). As a photoprotective mechanism, NPQ is conducive to decreases in ROS production in plant tissue (Oukarroum et al., 2015). In contrast to Zhefu802, Chl-8 showed higher NPQ, qE, and lower qI production of ROS under both moderate and high light treatments, which suggested that the higher NPQ in Chl-8 might be responsible for higher Pn in both light treatments via decreasing ROS levels. A large proportion of the NPQ was contributed by qE. In contrast to Zhefu802, Chl-8 had a higher qE and lower qI under both moderate and high light treatments. qE is major photoprotective component, whereas qI is mainly induced by the inactivation damage of PSII reaction centers and results from photoinhibition (Quaas et al., 2015; Ware et al., 2015). There were significantly lower levels of qI in Zhefu802 and Chl-8 under the moderate light treatment compared with the high light treatment. Chl-8 exhibited a lower decrease in Pn, F_v_/F_m_, and Φ_PSII_ under high light, which suggested that the higher NPQ in Chl-8 also favored the reduction of photodamage under high light. Chlorophyll fluorescence typically measures the top layers of chloroplasts in leaf, however gas exchange measures all layers. The measurements of NPQ may have their importance over-estimated in the moderate light. However, ASA significantly increased the Pn, Φ_PSII_, and F_v_/F_m_ values in the leaves of Zhefu802 under moderate light (Table 1). Interestingly, this beneficial effect was not observed in Chl-8. Though a substantial decrease in Φ_PSII_ was observed, the F_v_/F_m_ and Pn values increased only slightly when treated with ASA. In contrast, reducing NPQ caused by its inhibitor DTT decreased photosynthesis in both rice genotypes, in particular Chl-8 (Table 1). This result further supports the notion that NPQ is responsible for the high Pn observed in both rice genotypes.

It is well-known that qE is associated with the conversion of violaxanthin to zeaxanthin via the intermediate antheraxanthin, by the catalyst violaxanthin de-epoxidase (Niyogi et al., 1997, 1998) and the protonation of the PsbS protein (Li et al., 2002; Ware et al., 2014) under high light or gradually increasing light conditions. This was partly supported by our results whereby the induction of NPQ was determined by the expression of both *RVDE1* and *PsbS1* under moderate light intensity (Figures 10A,B). Previous studies have indicated that the expression levels of genes coding PsbS and violaxanthin de-epoxidase were consistent with protein levels (Bugos and Yamamoto, 1996; Bugos et al., 1999; Ishida et al., 2011; Zulfugarov et al., 2014). However, under moderate light, the content of zeaxanthin and the relative expression of *RVDE1* and *PsbS1* of Chl-8 were significantly higher than Zhefu802 (Figures 9, 10). Under high light, zeaxanthin, and *RVDE1*, rather than *PsbS1*, seemed to be primarily responsible for the induction of NPQ as there was little difference in the relative expression of *PsbS1* observed between the genotypes. The PsbS protein is necessary for the rapid induction of qE *in vivo*, acting as a sensor of the lumen pH such that qE can be rapidly activated (Betterle et al., 2009; Goral et al., 2012). Conversely, *in vitro*, the PsbS protein is dispensable since qE can be induced without the PsbS protein when the pH gradient is sufficiently high (Johnson and Ruban, 2011), suggesting that the direct protonation of LHCII or of other proteins in the thylakoid membrane can negate the necessity for PsbS. Interestingly, the presence of PsbS, but not zeaxanthin, has been found to offer better protection for PSII when plants are suddenly subjected to high light exposure (Ware et al., 2014). During the induction of NPQ, the organization of the PSII/LHCII supercomplexes located to the appressed thylakoids membrane were remodeled; this involved the structural reorganization of LHCII and organization of the thylakoid membrane (Anderson et al., 2012). The differentiation between varieties in the structure of the thylakoid membrane might be related to the induction of NPQ. The susceptibility of plant leaves to photoinhibition is linearly related to the degree of thylakoid membrane stacking. Plants with greater membrane stacking are more susceptible to photoinhibition and have higher D1 protein degradation (Anderson and Aro, 1994), and the degree of thylakoid membrane stacking in higher plants is regulated by light acclimation (Jiang et al., 2011). Zhefu802 was more susceptible to photoinhibition, which was demonstrated by the presence of thicker and more numerous stacks compared with Chl-8, irrespective of the light intensities imposed (Figure 11). Under high light, the number and thickness of stackings per chloroplast in Chl-8 was significantly less than under moderate light.

In summary, the Pn of Chl-8 was significantly higher than that of Zhefu802, although Chl-8 possessed significantly lower chlorophyll contents and a higher chlorophyll a:b ratio than Zhefu802. The differences in chlorophyll content and chlorophyll a:b ratio between the genotypes were correlated with differences in the ultrastructure of chloroplast. Both photorespiration and NPQ seemed to influence photoprotection and influenced the Pn of the genotypes under moderate light conditions. Irrespective of leaf color, NPQ could also improve the photosynthetic rate of rice under moderate light and alleviate photodamage under high light via a decrease in the generation of ROS. Both zeaxanthin content and the expression of *PsbS1* were involved in NPQ induction under moderate light, while only zeaxanthin content contributed to the induction of NPQ under high light.

## Author contributions

LT, GF, and XZ were involved in the formulation, experimental design, and write-up of the manuscript, while TC, XZ, BF, and CZ finalized the experiments involved in this paper. SP and XFZ provided scientific guidance for this research and critically revised the manuscript for important intellectual content. XZ and TC analyzed all data in this research. All authors listed in this paper approved the final manuscript.

## Funding

This work was funded by the National Natural Science Foundation of China (1501264, 31671619 and 31561143003), National Food Science and Technology Project (2016YFD0300208), Zhejiang Provincial Natural Science Foundation, China (LQ15C130003), the China National Rice Research Institute (2014RG004-4), the National System of Rice Industry (CARS-01-27) and the MOA Special Fund for Agro-scientific Research in the Public Interest of China (201203029 and 201203096).

### Conflict of interest statement

The authors declare that the research was conducted in the absence of any commercial or financial relationships that could be construed as a potential conflict of interest.

## Abbreviations

APX: Ascorbate peroxidase
ASA: Ascorbic acid
CAT: Catalase
DTT: Dithiothreitol
F_v_/F_m_: Maximum quantum efficiency of PSII
LHC: Light harvesting complex
MDA: alonaldehyde
NPQ: Non-photochemical quenching
PSII: Photosystem II
PSI: Photosystem I
POD: Peroxidase
qP: Photochemistry quench
ROS: Reactive oxygen species
SOD: Superoxide dismutase
qE: Energy quenching
qI: Photoinhibitory quenching
Φ_PSII_: Actual quantum efficiency of PSII.
