# Supplementary material for: Non-photochemical Quenching Plays a Key Role in Light Acclimation of Rice Plants Differing in Leaf Color
Source: Front Plant Sci. 2017 Jan 10;7:1968. doi: 10.3389/fpls.2016.01968 (PMC5222832; doi:10.3389/fpls.2016.01968)
Supplement: Supplementary file 1 [file DataSheet1.docx]

**Supplemental table 1** the primers used for qRT-PCR

| **Gene name** | **Gene ID** | **Forward** | **Reverse** |
| --- | --- | --- | --- |
| RVDE1 | Os04g0379700 | ACTTCTCCACGTTCATCAGGAC | TCTATCTCCTGCACTTCCCTGA |
| OSABA2 | Os04g0448900 | GTTCTAGGAGGAAACAGCACGA | TCCAATGCATCGTCATCCTCAA |
| PSBS1 | Os01g0869800 | CTGTTCGGCAGGTCCAAGAC | TTCAGCTGCGCCAGGATTC |
| beta-carotene hydroxylase1 | Os04g0578400 | CCGTATGGGCTGTTTCTTGG | CTCCGGTTGATTCTCGCAAG |
| beta-carotene hydroxylase2 | OS03g0125100 | TCACATGGACAAGTTCGAAGGT | CTCCTTCTCCAGCTCCTCAATC |
| beta-carotene hydroxylase3 | OS10g0533500 | CACCAGATACATCACACGGACA | CTTGATCTCCTTGTCCAGCTCC |
| Actin |  | AGATTAAGGTGGTCGCTCCA | AGAAGCACTTCCTGTGGACAA |
